# Opening Address Delivered before the Society of the Alumni of the Baltimore College of Dental Surgery, at the Second Annual Meeting, March 26th, 1850

**Published:** 1850-04

**Authors:** E. Townsend


### ARTICLE VI.

OPENING ADDRESS *delivered before the Society of the Alumni of the Baltimore College of Dental Surgery, at the Second Annual Meeting, March 26th,* 1850. By E. TOWNSEND, D. D. S.

MR. PRESIDENT AND GENTLEMEN:

The *science* of Dental Surgery, properly so called, is scarcely older than the oldest man in this assembly, and in this country it is almost as young as the youngest of its mature practitioners. Its origin, moreover, is not only a very recent, but it is also a very humble one; but, while we admit it is lowly born, we claim, also, that it belongs to a noble family. Surgery had its birth in the barber's shop, and the practice of medicine was, in its earliest age, the business of slaves. Our profession is only a younger child of the same lineage, and is already sturdily asserting its relationship in usefulness and honor.

If it was but the other day our department rose out of empiricism into the fair proportions of a regular science, the rapidity of its improvement has already achieved for it the honors of a maturer age. It is curiously true, that every useful art, and every practical science which serves and adorns our actual existence, is, in its main features, of very modern growth.

Chemistry and Geology have scarcely yet reached the ordinary length of a human life, and there is but little actually extant in any branch of the healing art, of a much older date; indeed, the arts and sciences which cover the whole domain of physical nature, and the inductive method of philosophy by which they are now cultivated, and destined to be ultimately perfected, have made their most considerable achievements quite within the memory of living men.

The honors of antiquity are therefore hardly worth disputing about, among the physical sciences; and the credit of the respective claimants will be better settled by their actual attainments, and energy of progress.

Twenty years ago Dentistry was practised as a secret art; its disciples evinced great exclusiveness, and carefully hid from each other the methods by which they attained, or thought they attained, any individual superiority. The profession was not then a fraternity; it had not the character of a liberal art; it had all the meagreness of a selfish individualism; its spirit was narrow and exclusive, and full of arrogance and pretence, and, while it thus encouraged all that is illiberal in rivalry, it hindered whatever is useful and noble in generous emulation. But this was not an essential or intrinsic meanness of the profession, it was the fault of its ignorance and inexperience; in a word, a fault inseparable from its infancy.

The earlier history of every branch of the healing art confesses similar blemishes, and all alike justly rest their present claims upon their present character. Now without forgetting the humility of the origin of Dental Science, or misapprehending the dignity of its destiny, or the actual distance which the profession now occupies from both, we may with a just pride point to the position which the labors of a faculty (of whom the greatest part survive) have given to it in the sisterhood of kindred sciences.

Its living legitimate professors, and the too early dead, who have bequeathed us their labors, and left us their memories to cherish, this honorable brotherhood of men have fairly and fully organised our profession in its lusty infancy, upon the principles of scientific truth, supplied it with the apparatus of orderly progress, elevated its standard of attainment, and secured for it the honors of a respectful recognition among the liberal and useful branches of art and knowledge, until we may now justly demand that it be judged and ranked according to its highest capabilities and aims, and not by the remaining and incongruous empiricism which still attaches to it. It is our proper business, it concerns our honor, we have made it our aim, and rigorously addressed ourselves to the task of putting our department of Surgical art into the highest position of honor and usefulness of which it is capable.

Dental Science has already in our country its regular periodical literature, and its standard elementary publications. It has its learned associations for the enlargement and mutual interchange of experience, and the diffusion of scientific discovery. It has in successful operation two *colleges* for the regular indoctrination of well educated and competent aspirants after solid usefulness and honorable fame, and *you*, the Alumni of the oldest and most famed of these, are associated for the purpose of permanently establishing those relations of mutual helpfulness and securing those reciprocities of professional and personal friendship which the generous spirit of fraternity in noble enterprise never fails to inspire. It is in the nature of mind to impart *most* liberally, its most valuable acquisitions, and to receive with an equally unselfish avidity all that the social commerce of intellect returns, just as light is transmitted and reflected from gem to gem in multiplied brilliancy, and as the vivifying rays of solar heat, flash from object to object till an equilibrium of the blessings give repose to the distributive impulse. It is only the lower relishes of the animal appetites that can enjoy a solitary feast. The raptures of the higher intellectual, and of the nobler, moral faculties are all found in a generous munificence, which emulates the "prodigality of heaven." This is not only the natural religion, but it is also a natural necessity of the intellect, for by a paramount law of human education it is ordained that by *giving* we shall receive, and in teaching we shall learn. Likely enough this may seem a paradox to the grudging earthling who would first separate himself from the community, violate the sympathies of general good, and then filch away from all around him to feed his own unthankful and unrepaying selfishness. To seize, to hide and hoard, are the only means of accumulation which the lowest instincts know, and it is not given to the shut soul and cavern heart to comprehend the divine policy of those high natures which acquire only to bestow, enjoy only what they spend, and lay up their chiefest treasures by giving them away. The "dungeon bosom and foggy head" understand not the wisdom of scattering the bread of life upon the waters which fertilize the field, and yield a manifold and rich harvest to both the reaper and the sower. A purblind selfishness never drops a seed into the soil until it has fenced in the enclosure, and fenced out all neighborhood of participation. Such natures learn their theory of solitary and exclusive property from insensate matter, and do not know that the economy of mind is based upon a community of good.

The light of a higher and truer philosophy, has directed you to the policy of professional association.

You understand the capabilities of union, (and in a solicitude for improvement, which appreciates alike the aim and the means,) you have pledged to each other all the mutual helpfulness of professional fraternity.

All manner of motives which a generous mind can feel, impel, and the happiest promises of good results invite you into community of effort, and interchange of advantages. You have come into the responsibilities of the profession at a period and under circumstances which demand your utmost efforts, as a duty and a debt, and may well awaken in you the strongest enthusiasm for scientific advancement.

The progress of practical knowledge is generally marked by a constantly increasing speciality of pursuit, and definite distinctness of drift, among the departments of its culture, and the healing art is already well divided by practice, into the several branches of Therapeutics, Surgery, and Obstetrics, and these again are rapidly resolving themselves into subordinate and distinct divisions. This is eminently true of the generic trunk from which we branch.

Surgery, involving, as it does, all the other departments of knowledge concerned about human life, its diseases and their treatment, and superadding as its peculiarity an art perhaps more difficult and delicate than them all, is seeking a distinct corps of professors and practitioners, for every well defined department of its diversified range. We have already the general operator, the Oculist, the Aurist, the Lithotomist, and the Dentist. *We* have chosen our function, and we must keep it at least abreast of the general advancement, and must make it deserve the world's confidence, and honorably answer its rising expectation.

Twenty years ago, two or three dentists sufficed for Boston, and the same number served for the great Valley of the Mississippi: now, there are about seventy; and at the same rate of increase, in the next twenty years there will be two hundred in Boston, and the forty to fifty who now find occupation in Cincinnati alone, will swell in the same proportion, and intimate the multitude that shall fill the great West. Just in proportion to our capability of meeting the natural want, will that want be felt, and every real advancement in attainment and professional ability will be rewarded with proportionate increase of demand, and enhanced appreciation of our department of the remedial art. I was engaged in the practice of Dental Surgery long before the establishment of *any* Dental College, and while I was, perhaps, more than ordinarily fortunate in preceptorial training and instruction, I was, at my outset in practice, without the accumulated light and knowledge of a distinguished faculty of professional instructors, such as the students of our colleges now possess.

My personal relations to the question, will therefore acquit me of all intended invidiousness, when I *insist* upon the obligation and the necessity of the most regular and general professional culture, that the novitiates in Dentistry can possibly obtain. I would not, in the mere indulgence of professional pride, nor in any overweening estimate of merely formal learning, nor in any disrespect or doubt of self-culture, prescribe expensive, embarrassing, and ceremonial compliances; but I would secure the highest qualifications for the men who are to practice our art in future, by the requirement of the most judicious and thorough training and instruction, that can be made available in education.

Mechanical skill and expertness of manipulation, do not make a dentist: though all operations absolutely depend upon dexterity of hand; for our profession is a scienceas well as an art. Like Statuary, it involves taste, as well as tact; like Surgery, it involves knowledge, as well as skill; and like remedial science in general, it demands systematic knowledge broadly based in anatomical facts, physiological laws, and medicinal agents. The acquirement of our professional learning, requires a scholar; its practice requires an artist; and its standing and social relations, *demand* a gentleman. Too much attention, therefore, cannot be given, too high importance cannot be accorded, to the means and methods of securing all these objects, and answering all these ends. Every branch of Surgery, which requires instrumental treatment, whether it be taking up an artery, amputating a limb, or filling, or extracting a tooth, depends upon manual dexterity, which can be had only on the terms of a thorough practical training; and no man can be tolerated in our profession, without such skill of fingers, and facility and precision of manipulation: but, on the other hand, it sinks into a mere handicraft, and loses all relation to its true objects and uses, in proportion as it is restrained in theory to mere mechanical expertness. Besides, there is a seductiveness in operations, and a display in sleight-of-hand, which, of itself, tempts indiscreet, incompetent, and dishonest men to indulge in them. This is as true in all the other branches of Surgery as in ours. It is a short way of settling the probabilities about a diseased limb, to sentence it to amputation; and the sacrifice of a diseased tooth may save some trouble, dispense with some science and skill, which the operator, perhaps, does not possess, and give an opportunity for a display of China ware, which, though only a piece of porcelain, actually looks like a human tooth. No where are the temptations to careless, hurried practice, greater than in the treatment of teeth, and no where does it deserve severer reprobation. The Dental Surgeon should remember that every time he extracts a tooth, he acknowledges against himself, or against his profession, or against both together, that he cannot cure, and therefore must mutilate. I repeat, it is the imperfection of our art, that we must extract at all.

It may be, indeed, in the very nature of things, impossible to avoid it in any conceivable state of knowledge, still that necessity will ever stand as a professional defect, and all progress, all real progress, consists in diminishing that necessity. I am not ashamed of my workmanship, nor do I refuse the credit it gives me, but the man who will teach me how to save a tooth, that I am now obliged to sacrifice, is my master in the science of Dentistry, without the proof of any other claim, and I gladly yield him the post of honor.

It is in this regard, in the preservation, the cure, the prevention of disease, that general and thorough medical science becomes available, and takes the rank I would assign it in Dental Surgery.

The teeth are a part, and no mean or very remote part of the general organization, their sensibility is delicate, though subdued, and their liability to disease, *itself* indicates their acute activity of life. In the proportion of this sensibility and liability, as well as of their functional importance, they are interlinked by active sympathies with the entire frame. Disease in distantly situated, but nearly related organs, effects them symptomatically, and their original derangements react again upon associated organs. The entire digestive apparatus is involved in these connections, by direct functional relation. Dyspepsia has been unsuccessfully treated, and teeth have been erroneously and needlessly extracted, in ignorance or neglect of this mutual dependency. Indeed, the man who has not remedied tooth-ache by emetics, cathartics, and other medicinal appliances, to the primæ viæ, has either had but little practice in dentistry, or deserved to have still less. I have taken an obvious instance to represent a principle.

There are many less familiar connections, and many, doubtless, quite unknown, in which general science would prove available in our branch of remedial practice. Besides, idiopathic affections of the teeth and gums, are themselves diseases, and their treatment supposes an acquaintance with the laws of life, both healthy and abnormal, and with hygienic and therapeutic principles. I would have every Dentist go in debt to the other branches of medicine for all they can confer upon him, and then repay it all by some worthy contribution of discovery in remedial treatment to the general stock.

There is not a truth of nature, not a principle in medical theory, not an appliance of practical Surgery, but may be light and power in the practice and the improvement of our branch. One would need to look back from the vantage ground of attainment still in advance of us, to decide which is the more important division of our general function; but first principles in advance of all experience assure us that all that enters into the art of prevention, preservation and cure, must take precedence of that which only mutilates, substitutes and replaces. And if we must needs indicate the specific department where the highest distinction is to be won, the profession would no doubt agree, that it is in the filling of teeth, and in the curative treatment of them.

It must not be understood that I would exaggerate any one particular or item in science or art so as to depreciate another. I do not balance one against another, but would endeavor to indicate the channel and direction of progress without assigning any difference of value or rank to the several tributaries upon each of which the great current equally depends; but, back of and above all that learning and skill can contribute lies a source of power and a spring of improvement, which is not over-valued when the *first* place in importance is assigned to it. I speak of professional morals; in which I include duty to our patients and *courtesy* to our brethren.

Under the first head, allow me remark that a fair reputation may be earned and enjoyed, and then abused, through indolence or greed of gain, or even through the pressure of crowding engagements, without any more culpable motive than the weakness of accepting more work than can be well and thoroughly executed. Through one or other of these causes a man who began with an honest purpose and an honorable ambition may be reduced to the meanness and selfishness of a mere jobber. Such a man, trusting to his established reputation, will save every moment of time from those nice points in a case which are at once troublesome and difficult, and, moreover, but little understood or appreciated by the patient, to give his attention where it pays better in credit and cash.

Now permit me to say, that fidelity to the trust reposed in the practitioner by the patient, carries with it an obligation so high, and so sacred, and so obvious too, that argument could only weaken and obscure it. As a matter of morality and professional honor, therefore, I let it rest on its own simplest statement. But the attention to minutiæ, the ardent struggle with difficulties, the patient effort and anxious study to remedy every form of disease, which spring from the impulses of sympathy and duty, are also the most reliable and most efficient precursors of professional improvement and scientific discovery; for it is not in the routine of ordinary, average, good-enough sort of practice, however well it may pass and answer for the present, that any real advancement of knowledge is made, but in that region of doubt and darkness which borders and bounds the cultivated and enlightened domain of the science.

Now, it needs all the impulses of an honest heart, in aid of the aspirations of an ambitious head, to thrust the busy practitioner out into this world of the unknown and unattempted, to grapple with the difficulties which challenge, while they seem to defy his skill. The temptations, as well as the real difficulties in the way of liberal enterprise are so great, that men need the full play of every motive which can influence to worthy effort. The delinquencies of unskillful men are of little comparative account. They are only concerned to conceal their ignorance and incapacity, and they are not dangerously able to effect it. But the skillful man is concerned to conceal his indolence and unworthiness, and *he* has the art to accomplish it. Let those of us who may deem our pretensions are best based, keep a wary eye upon our own conduct, lest we be in any way drawn aside or fall short of the fulfilment of our utmost duty to our patients and to the great interests of our calling. It is because I know how little the infinitely small and exquisitely nice points in our practice are understood and regarded, and how imperfectly they are rewarded, that I would fasten attention upon them as the avenues to larger and higher attainments, and nobler success. We must pardon the ingratitude of ignorance, we must consent to have our best endeavors overlooked and undervalued; we must generously sacrifice time and labor upon unconscious and unregarding objects, to live worthily of the high faith, and answer the noble hope of our professional position.

The knowledge, skill, and patience, for instance, required to prevent and remedy the deformity and eventual mischief of malposition in children's teeth, are not likely to be understood or appreciated by even the fondest and most discerning parents. If you will at once extract a couple of sound and unoffending molars or bicuspids, they will wait a year for the hoped for reformation in the incisors or cuspidati; but if you more dexterously "do little or nothing," as they regard it, every day or two for whole months in succession, they will scarcely know in the end whether art or nature has accomplished the cure. Well, what then? I say do your whole duty, for the grand reason that you owe it to yourself and to your profession, even if the patient is for any reason unworthy and undeserving.

*You* must not slight your office, because the party most interested in it regards it slightly. Remember that besides a self-approving pleasure, discovery and scientific enlightenment lies in the pathway of probity and patience, and it is moreover true also that lazy mediocrity is not more secure of its selfish ends, than honorable enterprise and aspiring enthusiasm are of their rewards; but if it were even otherwise, who would divorce the *man* from the profession, and so reduce the one into a sponge, and the other to the condition of a sordid trade!

From duty to our patients, I turn to the equally delicate and difficult subject of duty to our brethren. To them we owe justice, candor, and courtesy; we owe it to them in their own right; and for the sake of a common effort and aim, we owe to all, as well as need from all, a frank and respectful interchange of kindly offices.

Detraction is at once the vice of weakness, and the weakness and vice of strength. The consciously incompetent man has no other way of levelling himself into position, than by levelling or libelling better men down; and the arrogant man of fair talent, and unfair disposition, finds it convenient for increasing the supposed distance between his own and other men's abilities. Every man should know that he actually is not the best judge of his rival's rank, nor even of his own; or if it be impossible for conceit to yield this point, he ought to know that is extremely ungraceful to be at once his own trumpeter and his rival's accuser; nay, he must not be surprised if others should think that in doing this *double* duty, there is the double mixture of meanness and malignity. Coarse, open, outspoken detraction needs no special exposure, and against it, men worthy of our fellowship need no caution; but that phase of it which is practically implied in isolated pretensions, superiority of attainment and standing, which gathering up its skirts and mounting its stilts, denies the *fraternity* of the profession and dishonors it with an affectation of very general contempt—that assertion of exclusiveness which refuses to communicate, and denies its own need to receive from others—to say the least of such preposterous coxcombry, it is extremely unprofessional, for it ignores the very notion of a profession, and would keep Dental Surgery within the compass of a conjuror's cap, reduce its science to a system of lucky tricks, and its art into some sort of underworking sleight-of-hand.

To the *young* gentlemen before me, I may assume to speak, and I say to them, be guilty in no degree, no shade, no tint, or taint of such charlatanism. You will make discoveries, for that is the way to learn as much as other people already know, and I sincerely hope you will make discoveries quite beyond the present limits of our knowledge. If you do, if nature and education have made you great enough for that, don't be mean enough to run away into the dark with it; don't skulk out of good company to fatten on your good fortune, for recollect that you have borrowed as much from the liberality of others as your own genius will ever repay to the great world of science.

If there be anything revealed to you, give it to the light, that others may interpret, and test, and prove it. Recollect that the apostles of science have all things in common, and if you meanly secrete a part of the wealth which belongs of right to the general stock, you deserve to be carried out of the profession, like Ananias, *feet foremost.*

I have said enough to intimate pretty plainly, that *detraction,* or taking from and belying, and retraction, or drawing back and withholding, alike amount to contraction or puckering up, shrivelling and wilting, under the bitter blight of a selfish individualism.

Intimately connected with liberality in our personal relations, is the spirit of freedom in philosophical inquiry. As associates in study, and fellow-laborers in improvement, diversities of sentiment and conflict of views are to be expected.

Men are made unlike each other, that each may supply his peculiar gift to the common weal. A flat uniformity of opinion is neither profitable nor desirable. Let each, therefore, respect the individuality of every other, and so *entitle* himself to the liberties he claims for himself; and, above all, let us remember, that the legislation of majorities settles no question of science or fact.

Speculative truth and the resulting practice, lies within the domain of opinion, which is by nature free, and cannot be brought into bondage to any man, or any number of men. Moreover, it is not necessary that all questions should be settled and ended: it is necessary only that the truth should be known, and when every man has given his testimony faithfully he has done his whole duty; a step farther, and he is trespassing on the rights of others. Settling a question of opinion by authority, is only in fact *un*settling a great principle, by arresting inquiry, and forbidding future experience to illustrate and modify the past. Science is not a despotism, and its real cultivators are all equally freemen, and their liberty is as essential to the progression of truth, as it is to individual honor.

As we hope for improvement then, let us welcome change; and let us dispute no man's right of resolution who is able to overturn an idea, usage, or principle which is now in authority. Associations must not be used to crush out the freedom of the individual, and restrain the natural liberties of thought. There are enough securities for established truth, *in* its truth, without defending all we believe, with the jealousy of prejudice, and the fierceness of bigotry. The love of truth easily changes into passion for a creed: let us guard ourselves on that side, and so avoid the petrifaction of our opinions, which should be kept green and growing while there remains anything on earth to learn.

Restrained by the respect due to my audience, to whom I could not take the relation of teacher in any sense or particular, I have said but comparatively little on the art side of Dentistry; but enough, I hope, to show that it holds its due place in my estimation. It is indeed difficult to speak at large on this department, without becoming didactic, which the proprieties of my position forbid. In general, allow me to say, that dexterity and skill in the construction and application of instruments are as necessary to us as to the pianist, the sculptor, or the painter, and to acquire the power of transferring the highest thought into the closest correspondence of form and outward fact, is worth all the application which it costs, and all the time required in preparatory occupations. Without such skill of finger in some one, the brightest inspirations of genius would perish in the birth.

Dentistry is well divided into medical, surgical, and mechanical; both the latter branches are, in different ways, matters of handicraft, and they are the modes by which nearly all our knowledge comes into use. The education of the hand, and skill in metals and chemical processes, in carving, shaping, and symmetrical adjustments, will put a difference between two men, that no speculative superiority can overcome. All that can be said on this point is, that no art, trade, or profession, requires more skill in mere workmanship, or higher qualities of mind in its direction, than ours. Just as I have seen eloquence cripple for want of utterance, so I have seen science thrown into contempt from lack of manual skill. The Surgeon must undervalue nothing, from the boldest movement in a capital operation, down to the sharpening of an instrument, if he would be capable of all his duties; and a little experience will show him how silly it is to draw distinctions which have the affect of disqualifying him for anything he should know or do. Ignorance and awkwardness, are ignorance and awkwardness, whatever is the subject of them, and they are the misfortune and the shame of any man in any position which they injuriously affect.

Moreover, out of the confidence in one's hand and eye, which capital training induces a demeanor, assuring to the patient, and a feeling of self-balanced assurance to the operator arises, that is worth all the respectful consideration which we ask for them. The sympathies ascribed to mesmerism are more than realized in all kinds of surgical practice, and the steadiness and strength of felt competency, kindles the courage of the subject, while it gives nobleness and efficiency to the person upon whose conduct and capacity all the responsibilities rest.

Truly the skill which answers like a miracle to the inspiring volition, and again re-inspires the thought which it executes, is well worth having in the moment of trial. Besides there is in this matter of mechanism an element of beauty, which allies it closely to the highest offices of taste. An instance:—to set artificial teeth well, artistically, the Dentist must hit the correspondence and significance of their position and expression in the physiognomy.

The teeth bear a full share in the signs of character, and indicate it in their way as decidedly as eye or cheek or brow can do. This might seem more curious than clear or important, if it were not so well supported by facts on all sides. It is not questioned that forms correspond to uses in all mechanical functions, and a little reflection will show that the same rule is as absolute in the vital organization. Naturalists, such as Cuvier or Agassiz, can re-construct a fossil skeleton, it is said, from a single bone, if that bone be a decidedly characteristic one, as for instance, a bone of the wrist. The process is to infer the talons or fingers or hoops from the structure of the wrist; and from these the teeth; and from the teeth the digestive apparatus, and thence all the habits of the animal. Here the teeth are made the pivoted sign on which the whole solution turns. They have the whole character hieroglyphically recorded in their forms, number, attitudes, &c.

If then the differences among animals of different kinds are so ascertained with scientific certainty, it needs only deeper skill and quicker acuteness, to detect characteristic differences between the teeth of individuals of the same species. It is not to be expected that blunt indifferent minds, will either see or value such a point, but the difference between men, is in *favor* of those who see most and best and quickest.

But I must turn away from the attractive topics of our theme. It is too broad for discussion, even in the compactest forms of generalization, within the limits of my allotted time.

As the speaker honored by your selection, you had a right to expect from me the presentation of our common views and aims in some worthy form; but I had little to offer deserving your acceptance, besides my sympathy of sentiment and aspiration and my earnestness of purpose and effort. I love my profession, and feel far more the desire than the ability to promote its interests and honor.

The occasion, the audience and the circumstances claimed the utterance of the highest conceptions of our calling, its duties, principles and aims.

I have endeavored to give you my apprehension of such of them as I could press into the compass of this address. I wish, most unaffectedly, I wish that my contribution to the day's entertainment were worthy of this your second anniversary, worthy of the ideas and objects embraced by your organization, worthy of its high promises and prospects. I wish that it even came something nearer to the pitch and tone of my own conceptions, but I may hope, nevertheless, the inherent life of the subject matter has not been all lost in my imperfect forms of speech, and that you will find in it at least the inspiration of its intrinsic truth.

I close with a wish, that warms and deepens into the fervency and solemnity of prayer, for the interests, honor and improvement of our profession. The burden, the responsibilities and the honors, are justly divided amongst us, from the least even unto the greatest—to each according to his measure, to all in common, the largest range and the highest possibilities of attainment lie open.

Let us unite in a mutual pledge of fidelity to the trust, the duty and the hope.

