# Errata

**Published:** 1850-04

**Authors:** 


ERRATA.—We are requested to make the following corrections in Dr. Townsend's Address, published in another part of the present Number of the Journal. On third page of Address, 19th line of 2d paragraph, after solar heat, add *unresting*, and on 20th line of same paragraph, read *gives* for give. On 12th page, 1st paragraph, put a *comma* after I say. On 14th page, 2d line from the bottom of 1st paragraph, read *wonderworking* for underworking. On 15th page, 2d line from bottom, instead of resolution, read *revolution*. In 2d paragraph of 16th page, 3d line, omit, after I have said, the word *but*. On 17th page, 5th line from bottom of 1st paragraph, instead of affect, read *effect*. On 18th page, 12th line from top of 1st paragraph, read *hoofs* for hoops; and in 3d line from bottom of same paragraph, read *pivotal* for pivoted.

Some two or three other errors in punctuation have been pointed out, which, for want of room, we cannot correct.

